# Clinical and nutritional profile of children and adolescents with autism spectrum disorder in Brazil: a nationwide online survey

**DOI:** 10.1016/j.jped.2024.12.008

**Published:** 2025-05-31

**Authors:** Gabriele Gonçalves de Souza Corrêa, Fernanda Valente Mendes Soares, Zilton Farias Meira de Vasconcelos, Ana Carolina Carioca Costa, Adriana Duarte Rocha

**Affiliations:** Fundação Oswaldo Cruz, Instituto Nacional Fernandes Figueira, Unidade de pesquisa clínica, Rio de Janeiro, RJ, Brazil

**Keywords:** Autism spectrum disorder, Nutrition status, Eating and feeding disorder

## Abstract

**Objective:**

To characterize the clinical and nutritional profile of children and adolescents with ASD in Brazil and their eating problems.

**Method:**

This is a cross-sectional study using a national online survey, with a sample of 613 children and adolescents with ASD aged between 2 and 17 years. Data analysis consisted of descriptive analysis, followed by Pearson's chi-square test with a statistical significance of 0.05 and a 95% confidence interval.

**Results:**

Food allergy was reported by 33.8% of the participants, the most frequent being cow's milk (70.2%), among those who reported gastrointestinal problems, constipation was the most frequent (54.1%). The presence of pica was reported by 25% and food selectivity was present in 77.2%, with greater refusal of fruit, vegetables and pasty textures. Most of the participants do not have follow-ups with a nutritionist and 44.5% are on some special diet, excluding gluten/wheat (75.4%) and without casein/animal milk (76.1%). More than half of the participants did not eat fruit (50.6%), vegetables (68.1%), or leafy greens (83.6%) frequently. A positive correlation was found between food selectivity and gastrointestinal symptoms (p-value < 0.050); food allergy and gastrointestinal symptoms (p-value < 0.001) and pica and gastrointestinal symptoms (p-value < 0.001).

**Conclusions:**

The results of this study show changes in food consumption and increased risk of nutritional deficiencies for children and adolescents with ASD in Brazil.

## Introduction

Autism spectrum disorder (ASD) is a group of heterogeneous neurodevelopmental conditions characterized by early impairment in social skills, social communication and restricted and repetitive behaviors.^1^ It is caused by a combination of genetic and environmental factors, with a complex and multifactorial etiology,^2^ affecting 1 in 36 children according to data from the United States.^3^ In Brazil, to date, there is no prevalence data.

In addition to deficiencies in social communication and stereotyped behaviors, some comorbidities are often associated with ASD, such as disruptive behavior, gastrointestinal symptoms, and eating problems,^4^ including food selectivity, Pickiness/Ruminative Disorder and Avoidant/Restrictive Food Intake Disorder.^5^^,^^6^ Comorbidities can affect up to 74% of individuals with ASD, with a higher average number of comorbidities than their non-ASD siblings, and may be associated with pre- and post-natal exposures, such as fetal alcohol syndrome, severe prenatal infection, lead poisoning, and brain infections, and potential genetic risk factors.^7^

The incidence of chronic feeding problems can lead to multiple medical and developmental issues, such as malnutrition, growth failure, social deficits, and poor academic performance. Many factors can contribute to feeding problems in ASD, including a preference for routine and cognitive rigidity. However, sensory sensitivities are the most frequently cited, likely due to the atypical sensory processing associated with ASD.^8^ These feeding problems often impact nutritional status, causing metabolic changes and malnutrition, which can exacerbate early manifestations of abnormal social behaviors, sensory responsiveness, and obsessive behavior, contributing to a more severe clinical picture.^9^ Although information on these issues in children with ASD is limited, other studies with Brazilian children have found varying data, with food selectivity ranging from 34.4% to 53.4% in children with ASD and 13% presenting gastrointestinal symptoms. These children often have limited food choices, refuse to eat fruits and vegetables, follow rigid schedules and rituals, engage in minimal interpersonal interaction, and refuse to sit at the table.^10^^,^^11^

Dysfunctional immune responses can affect core behaviors in ASD.^12^ The National Health Interview Survey estimated that 9.6% of children with ASD have concomitant food allergies, compared to 4.0% of children without developmental disabilities.^13^ Food allergies can contribute to nutritional deficits^14^ and effective substitution diets are needed to adjust the nutritional status of these individuals.

This study contributes to the understanding of altered patterns of food consumption, gastrointestinal symptoms, and food allergies in children and adolescents with ASD in Brazil for future clinical guidelines and thus serves as a basis for measures to prevent nutritional deficiencies and manage comorbidities.

Considering these points, the aim of this study was to characterize the clinical and nutritional profile of children and adolescents with ASD in Brazil; describe their socioeconomic variables; analyze the clinical nutritional profile and morbidities and characterize the eating problems present in children and adolescents with ASD by describing the eating pattern and evaluating the correlation between food selectivity and gastrointestinal symptoms.

## Methods

This is a cross-sectional study, conducted through an online survey of national scope, including participants from all regions of Brazil. The questions were directed to parents or caregivers of children and adolescents with ASD, aged 2 to 17 years, who were exclusively fed orally, excluding individuals with RETT syndrome, trisomy 21, fragile X syndrome, Cornelia de Lange syndrome, tuberous sclerosis, and Prader-Willi syndrome.

The collection instrument was developed on the Google Forms® platform and widely disseminated and shared via the internet, through applications, social networks and national, state, and municipal associations, from December 2022 to February 2023, although it is not possible to accurately count the number of questionnaires sent.

The questionnaire, composed of 30 objective questions, was structured to identify data relating to socioeconomic profile, clinical and morbidity variables, nutritional variables, and food frequency, as described in Table 1.

**Table 1:** Questions present in the study questionnaire.

Socioeconomic profile	Education of those responsible
	Family income
	State they reside
	School attendance
	Age of the child/adolescent
Clinical and morbidity variables	ASD diagnosis
	Confirmed food allergy
	Medication use
	Gastrointestinal symptom/disorder
	Presence of pica – consumption of non-food substances
	Genetic alteration or syndrome
Nutritional variables	Follow-up with a nutritionist
	Special diet
	Presence of food selectivity
	Characteristics of food selectivity (color, texture, food groups
Food frequency	Healthy and unhealthy eating markers

The socioeconomic profile was composed of questions about the education of caregivers, family income, state they reside in, school attendance, and age of the child/adolescent. The level of education has a strong relationship with income according to previous studies performed in Brazil and can be used as a single indicator of socioeconomic level because educational stratification remains very strong in the country, thus building a more accurate income profile when combined with family income. The clinical and morbidity variables were determined through research in previous studies in this population and clinical practice of the researchers and the diagnosis of ASD was self-reported by their caregivers.

The use of dietary modifications is common in this population, thus the nutritional variables assessed the frequency of special diets and follow-up with a nutritionist, as well as gathered data on food selectivity and its characteristics.

Food frequency was estimated according to weekly, daily, and non-consumption parameters, with “frequent” being considered 4 to 7 days a week and “non-frequent” 0 to 3 days a week. The foods were divided into markers: Healthy food - fruit and natural juice, vegetables, leaves, cereals, legumes, animal proteins, and unhealthy food - fried food, sweets and candies, biscuits/crackers, packet snacks, soft drinks and artificial juices, instant noodles, and yogurt, according to the food frequency questionnaire of the National Demographic and Health Survey.^15^

The data was analyzed in SPSS 21.0, and Pearson's chi-square test was utilized to test associations between the variables food selectivity and gastrointestinal symptom; food allergy and gastrointestinal symptom; pica and gastrointestinal symptom, and calculating the p-value for this sample. The confidence level adopted was 95% for analyzing the critical value of the chi-square distribution, with a significance value of 0.05.

The study was approved by the Research Ethics Committee under CAAE: 65,372,822.7.0000.5269 IFF/FIOCRUZ.

## Results

Out of a total of 617 responses to the questionnaire, 613 met the inclusion/exclusion criteria. Four questionnaires were excluded because they had a genetic syndrome that had an impact on diet.

The socio-demographic data showed that most of the participants were of school age, lived in the southeast, had an income of between 2 and 5 salaries and went to a regular school, and most of their guardians had completed higher education (Table 2). The demographic distribution closely mirrors Brazil's data according to the Brazilian Institute of Geography and Statistics, with about 41% of the population residing in the southwest region, 8% in the midwest region, 26.9% in the northeast region, and 14.74% in the southern region. All regions of Brazil were included in the study. The level of education of caregivers differs from the national average, where only 19.2% have a college education, this may reflect the type of instrument used that uses internet access.

**Table 2:** Socioeconomic Profile of children and adolescents with ASD in Brazil.

Socioeconomic variables	**N**	**%**
Level of education of caregivers		
Incomplete elementary	5	0.8
Complete elementary	21	3.5
High school	174	29
College	347	57.8
Master's/PhD	52	8.7
I don't want to respond	1	0.2
Income (in minimum wages)		
Up to 2	104	17.3
2 to 5	180	30
5 to 10	120	20
>10	112	18.7
I don't want to respond	80	13.3
Region		
North	37	6.4
North East	104	17.9
Midwest	48	8.3
Southeast	309	53.2
South	83	14.3
Age		
Preschool (2 to 4 years old)	189	32
School (5 to 10 years old)	283	48
Teenagers (11 to 19 years old)	118	20
Attends school		
No	54	9
Yes, regular school	519	87
Yes, special school	24	4

Regarding the clinical and nutritional variables described in Table 3, 33.8% reported having some kind of food allergy, the most common being cow's milk allergy (70.2%). Among those who reported gastrointestinal problems, constipation was the most common (54.1%) and 49.2% use continuous medication, the most common being Risperidone. Most of the participants were not followed up by a nutritionist and just under half of them were on a special diet, most of which excluded gluten/wheat and casein/animal milk.

**Table 3:** Clinical and nutritional profile of children and adolescents with ASD in Brazil.

Variable	N	%
Food allergy		
No	388	66.3
Yes	198	33.8
Cow’s milk	139	70.2
Wheat/gluten	70	35.4
Egg	56	28.3
Peanut	31	15.7
Nuts	29	14.6
Fish/sea foods	37	18.7
Soy	33	16.7
Others	37	18.7
Gastrointestinal symptom		
No	281	47.2
Yes	314	52.8
Diarrhea	115	36.6
Constipation	170	54.1
Reflux	69	22
Abdominal pain	107	34.1
Nausea/vomiting	47	15
Others	3	1
Pica		
No	445	74.7
Yes	151	25.4
Use of medication		
No	301	50.8
Yes	291	49.2
Food selectivity		
No	136	22.8
Yes	461	77.2
Follow-up with a nutritionist		
No	393	66.2
Yes	201	22.8
Use of special diet		
No	329	55.5
Yes	264	44.5

In the analysis of food consumption (Table 4), the consumption of liver and leafy vegetables were the least frequent markers of healthy food and bread, biscuits or cookies were the most frequent markers of unhealthy food.

**Table 4:** Frequency of food consumption in adolescent children with ASD in Brazil.

Foods	Less common	Frequent
	N (%)	N (%)
Rice and pasta	211 (37%)	371 (63%)
Bread	363 (65.9%)	188(34.1%)
Bean	301 (53.35%)	264(64.2%)
Tubers	428 (75%)	143 (25%)
Chicken	413 (71.6%)	164(28.4%)
Leafy vegetables	480 (83.6%)	94 (16.4%)
Vegetables	396 (68%)	186 (32%)
Fruits	292 (50.5%)	286(49.5%)
Beef or pork	409 (70.6%)	170(29.4%)
Liver	530 (98.7%)	7 (1.3%)
Fish	531 (95.7%)	24 (4.3%)
Eggs	417 (73.3%)	152(26.7%)
Fried food	514 (90.7%)	53 (9.3%)
Sweets and treats	469 (82.6%)	99 (17.4%)
Biscuits or cookies	412 (72.7%)	155(27.3%)
Packaged snacks	519 (92.2%)	44 (7.8%)
Instant noodles	533 (97.1%)	16 (2.9%)
Soft drinks or artificial juices	500 (89%)	62 (11%)
Natural juice	379 (67%)	187 (33%)
Yogurt	508 (89.8%)	58 (10.2%)

A positive correlation was found between food selectivity and gastrointestinal symptoms (p-value < 0.050); food allergy and gastrointestinal symptoms (p-value < 0.001) and pica and gastrointestinal symptoms (p-value < 0.001).

## Discussion

The present study characterizes the clinical and nutritional profiles of children and adolescents with ASD in Brazil, detailing their socioeconomic variables, clinical nutritional profiles, and morbidities. It also describes the eating problems present in this population.

This study showed a high prevalence of eating problems, such as food selectivity (77.2%). Some international studies have also found similar results (46%−92%).^16^^,^^17^ However, in relation to the acceptance of textures, the children assessed preferred pasty or pureed textures, diverging from these findings where the majority of children and adolescents refuse pasty textures.

Food selectivity increases the risk of malnutrition, significant nutritional deficiencies, and possible gastrointestinal symptoms in children with ASD.^9^ The authors found a positive correlation between food selectivity and gastrointestinal symptoms (p-value < 0.050) in the sample of Brazilian children and adolescents with ASD, which may suggest a bidirectional relationship between food selectivity and gastrointestinal symptoms in this population, but the type of instrument used depends on the perceptions and interpretations of the guardians, which may be an information bias.

The mechanism by which selectivity can cause gastrointestinal symptoms may be linked to the restricted consumption of fruit and vegetables, thus reducing the availability of fiber and possibly triggering intestinal dysbiosis with a greater abundance of Enterobacteriaceae, Salmonella Escherichia/Shigella, and Clostridium XIVa^9^ but more studies are needed to screen for gastrointestinal symptoms in children and adolescents with ASD using a standardized assessment method such as the 2016 Rome IV criteria for functional gastrointestinal disorders, that provided a symptom-based management guide

Gastrointestinal symptoms are frequent comorbidities in individuals with ASD, with a 3 times higher risk of occurring than in their neurotypical peers, in this sample more than half of the participants reported some gastrointestinal symptom, with constipation being the most frequent (54%) followed by diarrhea (37%), abdominal pain (34%), reflux (22%) and nausea/vomiting (15%). Although constipation in children is one of the most common gastrointestinal disorders,^18^ the prevalence in children and adolescents in other Brazilian studies ranged from 14.7% to 36.5%,^19^ lower than that found in this study. The amount of fiber in the diet can be a predisposing factor for constipation and the low consumption of fruit and vegetables by children and adolescents with ASD can aggravate this condition.^19^

The assessment of gastrointestinal symptoms is especially complex due to the communication difficulties inherent to the disorder. These symptoms do not present in a typical way and can be identified by unusual behavioral changes,^9^ requiring parents, caregivers, and health professionals to be able to differentiate these behavioral changes when related to gastrointestinal symptoms, creating an additional challenge in the care of children and adolescents with ASD.

Pica, the ingestion of non-food items, can result in gastrointestinal parasites, lead poisoning, nutritional deficiencies, asphyxia, poisoning, sepsis, intestinal obstruction, or perforation, and can increase the risk of malnutrition, significant nutritional deficiencies and gastrointestinal symptoms in children with ASD, which can contribute to the presence of food selectivity.^9^^,^^20^ In the present study, 25.4% of guardians reported that children and adolescents consumed non-food items, similar to data found in a previous study where there was a prevalence of pica of 23.2% in children evaluated in the U.S.A.,^20^ to our knowledge, this is the first study to evaluate the prevalence of pica in children and adolescents with ASD in Brazil.

According to the National Health Interview Survey, food allergies are more prevalent in children with ASD. Data show that 9.6% of children with ASD^13^ had some type of food allergy, different from neurotypical children in the USA (8%)^21^ and Brazil (7.04%).^22^ In this study, 33% of caregivers answered that their children and adolescents had a confirmed food allergy, but due to the limitations of the instrument, clinical studies need to be performed to assess the prevalence of food allergies in children and adolescents with ASD in Brazil.

Food allergies can contribute to the presence of nutritional deficits^23^ and effective substitution diets are needed to adjust the nutritional status of these individuals, but only 22.8% of those caregivers reported follow-up with a nutritionist. In addition, 44.5% use some form of dietary modification, with the exclusion of gluten/wheat and cow's milk being the most common.

Over the years, several studies have aimed to assess whether the exclusion of milk protein and gluten could bring benefits to autistic people. A recent meta-analysis^24^ showed that a gluten and casein-free diet can reduce stereotyped behaviors and improve cognition in children with ASD, a result different from that found by a previous meta-analysis^25^ which found no benefits from the inclusion of a gluten and casein-free diet. It is well known that exclusion diets can cause nutritional deficiencies when they are not conducted by nutritionists to properly adjust micronutrients and macronutrients, so access to a professional nutritionist as part of a multi-professional team is essential to minimize symptoms related to food consumption, implement individualized strategies to correct nutritional deficiencies due to low consumption of fruits and vegetables and the use of allergen restriction diets.

Analyzing the frequency of consumption, there is a higher intake of rice (63.7% compared to beans (46.8%), but this is still lower than that found in studies with Brazilian children. In a study with a group of children aged 6 to 10 performed in Maringá-PR, it was found that 75.8% consumed rice and 75.7% beans every day,^26^ and an assessment of children at a municipal school in Belo Horizonte showed that around 97.8% of children in the same age group consumed rice and 95.6% beans every day.^27^ The fourth guideline of the Dietary Guidelines for the Brazilian Population proposes the daily consumption of beans and rice as a complete combination of proteins.^28^

There was frequent consumption of tubers and pumpkins by 25.1%, a finding contrary to the second guideline of the Food Guide,^28^ which recommends more frequent consumption of traditional Brazilian roots and tubers.

Animal protein consumption is below the prevalence of egg and/or meat consumption among children aged 24 to 59 months (88.9%), which can lead to protein malnutrition and low consumption of foods that are sources of essential nutrients for linear growth, such as proteins, essential fatty acids, vitamin B12, vitamin D, zinc, phosphorus, and selenium.^29^ Previous studies have reported inadequate amino acids in the plasma of autistic people and these nutritional deficiencies are related to the level of support of the individuals.^30^^,^^31^

Adequate consumption of fruits and vegetables is indicated for the prevention and control of chronic non-communicable diseases, which are responsible for the main causes of morbidity and mortality in Brazil. Among the participants who took part in the survey, 50.6% did not frequently consume fruit, a finding that differs from a study conducted in a population of children aged 0 to 5 in the state of Paraíba (25.1%)^32^ and from the National Study of Child Nutrition and Diet (ENANI)^33^

Less than half of the participants consumed vegetables and leafy vegetables frequently, which represents a lower intake of fiber and important micronutrients, especially iron, vitamins A, C and zinc.

In relation to the consumption of ultra-processed foods, the most frequent consumption (4 to 7 days) was of cookies and cookies (27.3%) followed by sweets and candies (17.5%), soft drinks/artificial juices (11%), yogurt (10.3%) fried foods (9.3%), packet snacks (7.9%) and lastly instant noodles. Data from the ENANI show that 88.8% of children aged 0 to 5 consumed ultra-processed foods on the day before the survey,^33^ but it was not possible to construct comparable indicators since in the ENANI the questions were related to food consumption on the previous day and in this survey they referred to weekly frequency.

The consumption of ultra-processed foods in children's diets is associated with an increase in weight gain, measures of adiposity, excess weight, early weaning, poor diet quality, metabolic alterations, diseases, and the consumption of chemicals from materials (plastic, paper, metal and glass) used in food packaging.^33^ High-fat consumption also represents an increased long-term risk for chronic health conditions among adults with ASD.^34^

The results of this study highlight the changes in food consumption and increased risk of nutritional deficiencies for children and adolescents with ASD in Brazil. Low consumption of food sources of protein, vitamins and minerals and high prevalence of food selectivity, gastrointestinal symptoms and food allergies can promote malnutrition and clinical and behavioral worsening.

The present study has some limitations that should be considered. The collection instrument used was an online questionnaire, without carrying out tests to prove some data, such as the presence of ASD, allergies, and genetic syndromes. This is a limitation of the collection instrument itself, however, necessary for greater coverage and inclusion of study participants. Data collection was carried out during the Covid-19 pandemic, so children and adolescents were not attending school during the dietary assessment period, which could impact the frequent consumption of some foods, especially rice and beans, typically offered in Brazilian schools. There is a lack of data regarding attendance in public and private schools, the study is limited to describing attendance in special or regular schools. Comparisons with previous articles may not reflect the reality of the typical school population, since these articles do not extract from the population consumption which children had ASD and which were typical. Future studies therefore should include similar analyses in order to avoid false positives in relation to the nutritional status of children with ASD compared to their healthy counterparts. The need for the internet to complete the questionnaire may limit access to low-income people and those with low education who need to read the questions presented.

Although the study obtained 613 responses, it was not possible to calculate the exact representativeness in relation to the total population of children and adolescents with ASD in Brazil. This is due to the lack of clinical studies and reliable epidemiological data that accurately estimate the prevalence of ASD in the country. This limitation of national data on ASD is a recurring challenge for research in the area. However, the present study ensured a diverse sample, with participants from all investigations in Brazil, to capture a comprehensive and varied view, which enriches the results of the study and contributes to the advancement of research on nutrition and childhood autism in Brazil.

Early assessment of signs of food selectivity, food allergies, and gastrointestinal disorders is necessary, since they are more frequent in this population to promote comprehensive care, respecting the particularities inherent in the disorder and developing clinical practices and public policies to promote health and quality of life. The authors suggest monitoring nutritional inadequacies and implementing nutritional strategies to expand the variety of foods consumed by children and adolescents with ASD, as well as routinely including the assessment of gastrointestinal problems in those with food selectivity or pica.

## Conflicts of interest

The authors declare no conflicts of interest.
